# *PLOS Mental Health*: Elevating the voices of lived experience to combat structural barriers and improve mental health globally

**DOI:** 10.1371/journal.pmen.0000053

**Published:** 2024-06-04

**Authors:** Karli Montague-Cardoso, Charlene Sunkel, Rochelle A. Burgess

**Affiliations:** 1 PLOS: Public Library of Science, Cambridge, United Kingdom; 2 South African Federation for Mental Health, Johannesburg, South Africa; 3 Movement for Global Mental Health, Johannesburg, South Africa; 4 UCL Institute for Global Health, London, United Kingdom

Our mental well-being is shaped by a host of interacting intrinsic and socio-political realities that are specific to us. Although we can relate to and empathize with others to varying degrees depending on our own understanding and experiences, we can only truly understand someone’s mental health and its causes, by listening to, and learning from them. The voice of lived experience is finally beginning to shape mental health research, treatments and policies. This influence is long overdue, but currently, efforts to incorporate lived experience perspectives are inconsistent. In 2022, the World Health Organisation (WHO) reported that 1 in 8 people worldwide live with a mental health condition [1]. This is likely to have been an underestimation for a myriad of reasons—and the same myriad of reasons may also account for the underrepresentation of diverse lived experience voices in the field as a whole.

Over the last decade or so, there has been an increase in the explicit encouragement of incorporating the lived experience perspective into all facets of the mental health field. For instance, the United Nations’ Sustainability Development Goal (SDG) 3, which is focused on good health and well-being [2], emphasizes the need for inclusive mental health policies and services that consider the perspectives and experiences of people with mental health conditions. The WHO has also been spearheading initiatives that are aimed at emphasizing the importance of involving people with mental health conditions in the development and implementation of mental health policies and services. The recent World Mental Health report [1] specifically discusses empowering those with lived experience and placing them as key stakeholders in transforming mental health care. Listening to those with lived experience will help us to fully understand both the vast and nuanced differences in experiences of individuals and communities worldwide. In particular, lived experience draws our attention to the realities and pains created by our social realities; poverty, political landscapes, violence, war, famine, racism and the colonial histories. Such factors demand that we engage in deep listening, so that mental health support can be developed to respond to both causes and consequences of social and structural determinants. It is only through the honouring of lived experience that we can challenge the status quo and break down the structural barriers that are preventing far too many from accessing the mental health care that they need. From accessing their basic human rights. We need to incorporate their perspectives on the same level and with the same enthusiasm held for researchers and professionals.

We must also react, as a priority, to the causes of discrepancies in access to mental healthcare and societal attitudes globally in order to improve well-being and access to treatments, as well as reduce stigma. Achieving this not only requires listening to the lived experience perspective but is also dependent on consistent cross-talk between different mental health related disciplines. Getting everyone around the same table, from researchers, to clinicians, to policy makers and those with lived experience will ensure that we are able to steer the field towards a direction that eliminates stigma and barriers to care and well-being. *PLOS Mental Health* starts its journey during a time when millions are experiencing trauma and challenges that make us question the workings of the human race. As we progress as a journal, we will adapt as and when needed in order to authentically serve mental health communities in the ways that they need.

We are excited that our launch content covers a range of topics, is cross-disciplinary, and comes from a diverse pool of authors in terms of region and seniority: a collection that is truly reflective of the fundamentals of our journal’s mission. Lived experiences feature heavily among our first Research Articles. This includes an analysis of the role of friendships in the well-being of adolescents with Attention Deficit Hyperactivity Disorder, as well as an analysis of research priorities of those with lived experiences in Australia, and the perspectives of clinicians when dealing with suicide risk management. Our commitment to diversity of contexts and communities is reflected within articles focusing on the understanding and management of depression in those with HIV in Ghana and Uganda as well as an analysis of psychoactive substance use in secondary school students in Cameroon. Aside from these articles, our first publications also include Occupational Mental Health, Underlying Mechanisms of Mental Health Conditions, Public Mental Health and more.

Our first Opinion pieces tackle some high priority areas for *PLOS Mental Health*. ‘Well-being and Emotion’ Section Editor Sidarta Ribeiro and his colleagues discuss the importance of managing mental health holistically and challenge the current overreliance on medication as the ‘go-to’ approach in the clinic. ‘Cognition and Mental Health’ Section Editor Christian Beste also challenges common perspectives of addiction and argues that framing some addictive traits as positive and using those when managing addiction, could be beneficial. Our other two Opinion pieces shine a spotlight on lived experiences and how those vary globally. Academic Editor Giovanni de Girolamo and his colleagues discuss language barriers for non-native speakers of English, especially in the context of those working in mental health research and how we can work to reduce that barrier. Finally, Lakshmi Gopalakrishnan considers regional differences in understanding and supporting perinatal depression and priorities for moving forward.

As we continue to publish content and elevate voices that are representative of *all* communities, we hope to play a role in sparking the urgent changes needed to break down the structural barriers that have been holding back many for so long. At *PLOS Mental Health*, we refuse to be a passive or stagnant voice. Our first content is our flag in the stand–as we strive to challenge and change, and play a role in improving mental health and well-being globally.
